# Prenatal Exposure to Polycyclic Aromatic Hydrocarbons, Benzo[*a*]pyrene–DNA Adducts, and Genomic DNA Methylation in Cord Blood

**DOI:** 10.1289/ehp.1104056

**Published:** 2012-01-17

**Authors:** Julie B. Herbstman, Deliang Tang, Deguang Zhu, Lirong Qu, Andreas Sjödin, Zheng Li, David Camann, Frederica P. Perera

**Affiliations:** 1 Columbia University Mailman School of Public Health, Department of Environmental Health Sciences, New York, New York, USA; 2 Centers for Disease Control and Prevention, Atlanta, Georgia, USA; 3 Southwest Research Institute, San Antonio, Texas, USA

**Keywords:** adducts, epigenetics, methylation, polycyclic aromatic hydrocarbons, prenatal, umbilical cord blood

## Abstract

Background: Polycyclic aromatic hydrocarbons (PAHs) are carcinogenic environmental pollutants generated during incomplete combustion. After exposure and during metabolism, PAHs can form reactive epoxides that can covalently bind to DNA. These PAH–DNA adducts are established markers of cancer risk. PAH exposure has been associated with epigenetic alterations, including genomic cytosine methylation. Both global hypomethylation and hypermethylation of specific genes have been associated with cancer and other diseases in humans. Experimental evidence suggests that PAH–DNA adduct formation may preferentially target methylated genomic regions. Early embryonic development may be a particularly susceptible period for PAH exposure, resulting in both increased PAH–DNA adducts and altered DNA methylation.

Objective: We explored whether prenatal exposure to PAHs is associated with genomic DNA methylation in cord blood and whether methylation levels are associated with the presence of detectable PAH–DNA adducts.

Methods: In a longitudinal cohort study of nonsmoking women in New York City, we measured PAH exposure during pregnancy using personal air monitors, assessed PAH internal dose using prenatal urinary metabolites (in a subset), and quantified benzo[*a*]pyrene–DNA adducts and genomic DNA methylation in cord blood DNA among 164 participants.

Results: Prenatal PAH exposure was associated with lower global methylation in umbilical cord white blood cells (*p* = 0.05), but global methylation levels were positively associated with the presence of detectable adducts in cord blood (*p* = 0.01).

Conclusions: These observations suggest that PAH exposure was adequate to alter global methylation in our study population. Additional epidemiologic studies that can measure site-specific cytosine methylation and adduct formation will improve our ability to understand this complex molecular pathway *in vivo*.

Polycyclic aromatic hydrocarbons (PAHs) are carcinogenic environmental pollutants resulting from incomplete combustion that are commonly found in tobacco smoke, ambient and indoor air, and charbroiled foods. After exposure, these compounds are metabolized to form phenolic products and reactive epoxides, which have the capacity to bind to DNA, forming PAH–DNA adducts (Whyatt et al. 1998). PAH–DNA adducts increase the likelihood of gene mutations and have been associated with various forms of cancer (Kriek et al. 1998). PAHs can cross the placenta, and previous studies have suggested that the developing fetus may be 10 times more susceptible than the mother to PAH-induced DNA damage (Perera et al. 2005).

PAH exposure has also been associated with epigenetic alterations, including genomic cytosine methylation. Benzo[*a*]pyrene (BaP), a five-ring mutagenic PAH, has been shown to disrupt DNA methylation patterns in experimental systems (Sadikovic et al. 2007; Wilson and Jones 1983). Additionally, global DNA hypomethylation is associated with genome instability and subsequent cancer risk (Hoffmann and Schulz 2005; Wilson et al. 2007). Early embryonic development may be a particularly susceptible period for epigenetic dysregulation as a consequence of environmental exposures, because DNA synthesis rates are high and DNA methylation patterns are being established (Dolinoy et al. 2007).

Prenatal exposure to PAHs may result in both increased PAH–DNA adducts and altered DNA methylation, and both mechanisms have been associated with increased cancer risk (Pfeifer 2006). In addition, experimental studies suggest that PAH-induced DNA adducts may preferentially form at guanine residues near methylated cytosines (Chen et al. 1998; Denissenko et al. 1996; Tretyakova et al. 2002; Weisenberger and Romano 1999).

In this epidemiologic study, we sought to determine whether prenatal exposure to PAHs is associated with altered global methylation in umbilical cord white blood cells (WBCs) and to determine whether methylation is associated with the presence of detectable PAH–DNA adducts.

## Materials and Methods

*Study design.* The Columbia Children’s Center for Environmental Health (CCCEH) Northern Manhattan Mothers and Newborns Study is a longitudinal cohort study comprising African-American and Dominican women who were recruited in the prenatal clinics of New York Presbyterian Medical Center, Harlem Hospital, or their satellite clinics, as previously described (Perera et al. 2002). Eligible women included those who did not smoke or use illicit drugs; were 18–35 years of age at time of delivery; registered in the prenatal clinics by the 20th week of pregnancy; were free of diabetes, hypertension, and reported HIV infection; and had resided in northern Manhattan or the South Bronx neighborhoods of New York City for at least 1 year. Eligible women who gave informed consent were considered “initially enrolled.” Women who completed 48-hr personal prenatal air monitoring during the third trimester of pregnancy and the collection of maternal and/or umbilical cord blood were considered “fully enrolled” (Perera et al. 2003).

**Figure 1:**
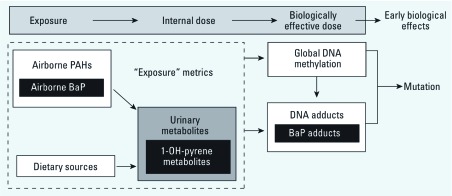
Conceptual association between exposure measures and biomarkers along the toxicologic paradigm. Indicators of exposure include airborne BaP and other PAHs and dietary sources of PAHs. Biomarkers of internal dose include 1-OH-pyrene metabolites. Biomarkers of biologically effective dose include global DNA methylation and PAH–DNA adducts.

For this study, we randomly selected 164 participants from the CCCEH cohort of 725 women with stored cord blood DNA, half with prenatal PAH exposure levels above and half with exposures below the population median (PAHs, including pyrene, 5.314 ng/m^3^; PAHs, not including pyrene, 2.265 ng/m^3^). In addition to PAH exposure, we considered PAH-urinary metabolites and BaP–DNA adducts as indicators of internal dose and biologically effective dose, respectively (Figure 1).

*Prenatal personal PAH assessment.* During the third trimester of pregnancy, personal monitoring was carried out as previously described (Perera et al. 2003, 2006). Vapors and particles ≤ 2.5 μg in diameter were collected on a precleaned quartz microfiber filter and a precleaned polyurethane foam cartridge backup. The samples were analyzed at Southwest Research Institute (San Antonio, TX) for pyrene, benz[*a*]anthracene, chrysene, benzo[*b*]fluoranthene, benzo[*k*]fluoranthene, BaP, indeno[1,2,3-*cd*]pyrene, dibenz[*a*,*h*]anthracene, and benzo[*g*,*h*,*i*]perylene as described by Tonne et al. (2004). For quality control, each personal monitoring result was assessed for accuracy in flow rate, time, and completeness of documentation. All of the 164 subjects had samples of acceptable quality.

*Measurement of PAH metabolites.* We measured PAH metabolite levels in the urine collected prenatally from a subset of participants in our present study sample (*n* = 87). The availability of urinary metabolite measurements was dependent on the participant’s willingness to donate a urine sample and the quantity of urine donated in relation to the requirements of the assay. Each subject’s prenatal urine sample was analyzed at the Centers for Disease Control and Prevention (Atlanta, GA) for a suite of 10 PAH metabolites as previously described: 2-, 3- and 9-hydroxyfluorene, 1- and 2-hydroxynaphthalene, 1-, 2-, 3-, and 4-hydroxyphenanthrene, and 1-hydroxypyrene (Li et al. 2006). Analytical determination was conducted by using enzymatic deconjugation, followed by automated liquid–liquid extraction, and quantified by gas chromatography/isotope dilution high-resolution mass spectrometry. The limit of detection (LOD) was defined as the higher of either the method blank LOD (three times standard deviation of method blanks after subtracting the average blank) or the instrument LOD (lowest point on the calibration curve having a signal more than three times the signal-to-noise ratio). Values below the LOD were recoded as half the LOD. PAH metabolites were detected in all samples except 3-hydroxyfluorene (eight samples < LOD), 1-hydroxyphenanthrene (one < LOD), 3-hydroxyphenanthrene (two < LOD), and 4-hydroxyphenanthrene (three < LOD). To control for urine dilution, we adjusted metabolite concentrations for urine specific gravity levels, which were measured using a handheld refractometer (model PAL-10-S-P14643C0 urine specific gravity refractometer; TAGO USA Inc., Bellevue, WA). As described in previous publications, we used urinary specific gravity rather than urinary creatinine to account for urinary dilution, because urinary creatinine may provide a biased estimate of dilution in asthmatics (and ~ 25% of our subjects are asthmatics) (Miller et al. 2010).

*Analysis of global DNA methylation.* DNA isolated from umbilical cord blood leukocytes was analyzed for global DNA methylation using the Methylamp^TM^ Global DNA Methylation Quantification Kit (Epigentek Group Inc., Farmingdale, NY). The assays were run on four 96-well plates, and all samples (*n* = 164) were run in duplicate on the same plate. This methodology quantifies the methylated fraction of DNA using an ELISA-like reaction. The proportion of methylated DNA in the full sample is determined by plotting the intensity of the optical density generated from the reaction compared with the standard curve produced using a methylated DNA control. In the samples analyzed in this study, the coefficient of variation between replicates was 16%.

*BaP–DNA adducts.* BaP–DNA adducts were analyzed in umbilical cord blood samples with sufficient DNA quantities (*n* = 152, 93% of the study sample). BaP is widely used as a representative PAH because concentrations of individual PAHs in the urban setting are highly correlated; therefore, we used BaP–DNA adducts as a proxy for PAH–DNA adducts (Perera et al. 2004). We analyzed BaP–DNA adducts in extracted WBC DNA using a high-performance liquid chromatography–fluorescence (HPLC-F) method, which detects BaP tetrols released after hydrolysis of DNA adducts during the sample preparation (Alexandrov et al. 1992). The method has a coefficient of variation of 12% and a lower LOD of 0.25 adducts per 10^8^ nucleotides.

*Questionnaire-based information.* We obtained information about demographic characteristics based on responses to an interviewer-administered questionnaire at baseline during prenatal visits. In addition, we used questionnaire-based information to estimate dietary exposure to PAHs by quantifying the consumption of smoked, fried, broiled, and barbecued meats. We also estimated exposure to environmental tobacco smoke (ETS) using a combination of questionnaire-based information (whether or not the mother cohabitated with a smoker during pregnancy) and/or whether cotinine levels measured in umbilical cord blood > 1 ng/mL (Benowitz et al. 2009).

*Statistical analysis.* The total PAH level in air was calculated as the sum of the eight PAHs measured (excluding pyrene). Pyrene and BaP in air were analyzed separately. Prenatal air PAH levels were natural log transformed when modeled as continuous variables or were divided at the median for categorical analyses. For the DNA methylation analyses, we used the average of the proportion of methylation generated from the duplicate wells, unless the duplicate result did not meet our quality assessment and control criteria. In five cases, we used the proportion of methylation generated from one quantifiable well. Genomic methylation was natural log transformed. One sample with no detectable methylation was assigned the lowest measurable methylation value in our sample (0.08 ng/100 ng total DNA) divided by the square root of 2. BaP–DNA adducts were dichotomized as either detectable or not detectable; there were 12 participants for whom BaP–DNA adduct measurements were not available. We used multivariate linear regression models to estimate the associations between prenatal PAH exposure (measured in both air and urine) and methylation. We used multivariate logistic regression models to estimate the associations between methylation and the presence or absence of adducts and to explore whether methylation alters the association between prenatal PAH exposure (in air and urine) and the presence of adducts (Baron and Kenny 1986). Covariates were considered potential confounders if related to genomic methylation based on previous reports (Terry et al. 2008), including maternal age, ethnicity, marital status, education, annual household income, child’s sex, and mother’s parity. Covariates were included in multivariate models as potential confounders only if they were associated with both the independent and dependent variables at *p* < 0.15. In all other cases, an α of 0.05 is considered statistically significant. Analyses were conducted using STATA/SE (version 11.0; StataCorp, College Station, TX) and the ggplot2 package (Wickham 2009) in R (version 2.14.1; R Foundation for Statistical Computing, Vienna, Austria).

## Results

The distributions of PAH measurements in air and urine, BaP–DNA adducts, and DNA methylation in our study population are described in Table 1. Median BaP and total PAH concentrations were 0.24 and 2.47 ng/m^3^, respectively. BaP–DNA adducts were detected in half of all participants. DNA methylation ranged from 0.06 to 5.42 ng/100 ng total DNA. Demographic characteristics in this study sample were similar to those of the underlying cohort (Table 2). Two-thirds of the participants were Dominican, and one-third were African American. Most participants were unmarried and had an annual household income < $30,000. Consistent with the study sample selection criteria, half (49.4%) of the study sample had prenatal air PAH levels above the population median of 2.3 ng/dL, and half (50.6%) had air PAH levels below the median (Table 2). The proportion of participants with detectable BaP–DNA adducts in this sample (50%) was significantly lower than in the underlying cohort (67%).

**Table 1:** Distribution of PAH exposure in air and urine, BaP–DNA adducts, and DNA methylation in our study population.

Percentile					Geometric mean (GSD)
Exposure variable	n	5th	25th	50th		75th	95th
PAHs in air (ng/m3)a										
Benz[a]anthracene		164		0.05	0.13	0.21	0.40	0.84		0.23 (2.30)
Chrysene		164		0.05	0.15	0.27	0.46	1.09		0.27 (2.39)
Benzo[b]fluoranthene		164		0.11	0.20	0.46	0.85	2.08		0.45 (2.72)
Benzo[k]fluoranthene		164		0.04	0.05	0.10	0.20	0.54		0.11 (2.70)
BaP		164		0.04	0.12	0.24	0.62	1.54		0.27 (3.26)
Indeno[1,2,3-cd]pyrene		164		0.05	0.19	0.41	0.89	2.94		0.41 (3.25)
Dibenz[a,h]anthracene		164		0.02	0.04	0.05	0.06	0.10		0.05 (1.73)
Benzo[g,h,i]perylene		164		0.12	0.34	0.67	1.35	4.66		0.69 (3.27)
Pyrene		164		1.16	1.70	2.62	4.94	11.18		2.98 (2.14)
Total PAHsb		164		0.64	1.28	2.47	5.05	13.53		2.66 (2.71)
PAHs in urine (ng/L urine)b										
2-Hydroxyfluorene		87		109.8	176.4	238.9	335.7	879.4		257.6 (1.94)
3-Hydroxyfluorene		87		11.8	27.7	40.8	68.0	167.8		44.6 (2.39)
9-Hydroxyfluorene		87		266.3	408.2	627.5	987.9	1957.0		657.9 (1.96)
1-Hydroxynaphthalene		87		404.9	958.4	1870.2	3048.9	10887.0		1848.7 (2.83)
2-Hydroxynaphthalene		87		961.0	1758.5	2814.3	4573.2	18861.6		3117.3 (2.47)
1-Hydroxyphenanthrene		87		110.6	175.5	251.0	440.5	1029.2		280.9 (1.98)
2-Hydroxyphenanthrene		87		42.2	68.1	110.7	199.1	372.0		118.2 (2.04)
3-Hydroxyphenanthrene		86		28.6	49.0	79.8	152.7	298.8		84.3 (2.17)
4-Hydroxyphenanthrene		83		17.3	33.1	54.1	109.3	261.9		59.8 (2.30)
1-Hydroxypyrene		87		53.9	105.2	154.1	214.6	472.1		152.8 (2.0)
BaP-DNA adducts (adducts/108 nucleotides)		152		ND	ND	ND	0.34	0.52		0.32 ±1.04c
DNA methylation (ng/100 ng total DNA)		164		0.31	0.93	1.34	1.81	3.28		1.24 (2.00)
Abbreviations: GSD, geometric standard deviation; ND, not detected. aSum of all PAHs measured (except pyrene). bSpecific gravity adjusted using the following formula: PAH concentration = PAHs × [(1.019 – 1)/(Specific gravity – 1)], where constant (1.019) refers to the median specific gravity measure observed in this cohort. cMean ± SD.										

**Table 2:** Characteristics of subjects included in this analysis and the underlying cohort [n (%)].

Characteristic	Present analysis (n = 164)	Full cohort (n = 727)
Maternal age (years)				
< 20		28 (17.1)		121 (16.7)
20–24		64 (39.2)		289 (39.8)
25–29		48 (29.3)		176 (24.2)
≥ 30		24 (14.6)		140 (19.3)
No. missing		0		1
Ethnicity				
Dominican		113 (68.9)		473 (65.1)
African American		51 (31.1)		254 (34.9)
No. missing		0		0
Marital status				
Not married		126 (77.3)		529 (72.8)
Married/cohabitating		37 (22.7)		194 (26.7)
No. missing		1		4
Maternal education				
< High school		46 (28.8)		257 (36.0)
Completed high school		66 (41.2)		285 (40.0)
> High school		48 (30.00)		171 (24.0)
No. missing		4		14
Annual household income				
< $10,000		66 (40.7)		303 (42.2)
$10,000–30,000		65 (40.1)		277 (38.6)
> $30,000		31 (19.1)		138 (19.2)
No. missing		2		9
Child sex				
Male		83 (50.6)		351 (48.3)
Female		81 (49.4)		376 (51.7)
No. missing		2		9
Parity				
Nulliparous		42 (25.6)		175 (24.2)
Multiparous		122 (74.4)		548 (75.8)
No. missing		0		4
Total PAHs				
> Median (2.3 ng/m3)		81 (49.4)		344 (49.9)
< Median		83 (50.6)		343 (50.1)
No. missing		0		40
BaP–DNA adductsa				
Nondetected		80 (50.0)		218 (33.4)
Detected		80 (50.0)		434 (66.6)
No. missing		4		75
aDifference between study sample and population sample significant at p = 0.01.				

*Indicators of PAH exposure.* We used airborne PAH exposure and PAH urinary metabolites measured in maternal urine collected prenatally from a subset of 87 women to estimate prenatal PAH exposure and internal dose, respectively. Demographic characteristics were not associated with PAH exposure indices, with a few exceptions: African Americans were significantly more likely than Dominicans to have high geometric mean levels of 1-hydroxynaphthalene (3117.1 vs. 1497.5 ng/L urine), 2-hydroxyfluorene (337.1 vs. 231.2 ng/L urine), and 4-hydroxyphenanthrene (80.3 vs. 53.0 ng/L urine). Women with higher income had lower levels of 1-hydroxypyrene: Women with annual household incomes < $10,000 had geometric mean 1-hydroxypyrene concentration of 187.8 ng/L urine, compared with 137.2 and 130.2 ng/L urine among women making $10,000–30,000 and > $30,000, respectively.

Mothers exposed to ETS during their pregnancy were, on average, younger, more likely to be African American than Dominican, less likely to be married, and more likely to have lower annual household income than were mothers who were not ETS exposed (data not shown). Those with ETS exposure had higher levels of 3-hydroxyfluorene (56.2 vs. 37.5 ng/L urine, *p* = 0.04). African Americans tended to consume more PAH-containing meats than did Dominican participants. However, our index of PAHs from smoked, broiled, fried, or barbequed meat was not correlated with any of our PAH metabolite measures (*r* = –0.11 to 0.19).

The total PAH level in air was positively correlated with pyrene and BaP (Pearson’s *r* = 0.69 and 0.96, respectively), and individual PAH metabolites were also correlated with one another (*r* = 0.30–0.89). The total airborne PAH level was only moderately correlated with 2-hydroxyfluorene, 2-, 3-, and 4-hydroxyphenanthrene, and 1-hydroxypyrene (*r* = 0.20–0.26) and was not correlated with the other measured PAH metabolites. Pyrene in air was moderately correlated with all measured metabolites (*r* = 0.20–0.49), except for the naphthalene metabolites.

*PAH exposure and DNA methylation*. Using univariate linear regression, we found that cord blood global methylation was lower (*p* < 0.01) among African Americans (geometric mean = 1.04 ng/100 ng total DNA) than among Dominicans (1.35 ng/100 ng total DNA). However, no other demographic variables were significantly associated with global methylation levels.

**Figure 2:**
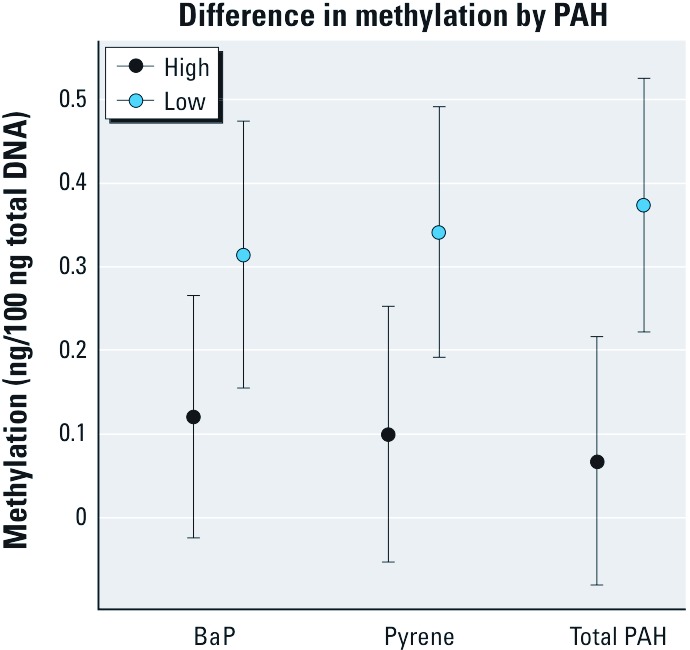
Global DNA methylation in cord blood according to prenatal air PAH exposure (based on univariate analyses). “High” and “Low” represent the geometric mean methylation (and 95% CIs) for those with prenatal concentrations above and below the median, respectively.

Natural log–transformed methylation was negatively associated with the natural log of prenatal air PAH exposure [β = –0.11; 95% confidence interval (CI): –0.21, 0.00; *p* = 0.05]. Associations were similar for pyrene (β = –0.18; 95% CI: –0.32, –0.04; *p* = 0.01) and for BaP (–0.09; 95% CI: –0.18, 0.00; *p* = 0.06). Methylation levels among participants with BaP, pyrene, and total PAHs above the median (“high”) were lower than methylation levels among those with BaP, pyrene, and total PAHs below the median (“low”) (*p* = 0.08, 0.03, and 0.01, respectively) (Figure 2). Participants exposed to prenatal ETS had levels of methylation similar to levels in those who were not exposed (1.25 vs. 1.24 ng/100 ng total DNA, *p* = 0.90). When ethnicity was added to the multivariate linear regression models to assess confounding, none of these associations (β-coefficients) changed, leading us to conclude that the associations were not confounded by ethnicity.

Participants with PAH urinary metabolite data were similar to the rest of the sample in terms of total air PAH concentrations and proportion of detectable DNA adducts, but they had significantly higher average global DNA methylation (geometric mean of 1.00 vs. 1.51 ng/100 ng DNA, *p* < 0.01). In this subset of participants (*n* = 87), a slightly positive but nonsignificant association was observed between metabolite levels and global DNA methylation when two heavily influential observations were removed (all *p*-values > 0.10) (data not shown).

*PAH exposure and BaP–DNA adduct formation.* No association was observed between any of the covariates presented in Table 2 and the presence of BaP–DNA adducts (*p* > 0.10), except for parity: Nulliparous women were less likely to have detectable adducts (*p* = 0.06) than were multiparous women. Detectable adducts were not associated with any of the PAH exposure indicators, including all measures of air exposure, ETS, dietary PAHs from meat, and (in the subset for whom they were measured) PAH metabolites (data not shown).

**Figure 3:**
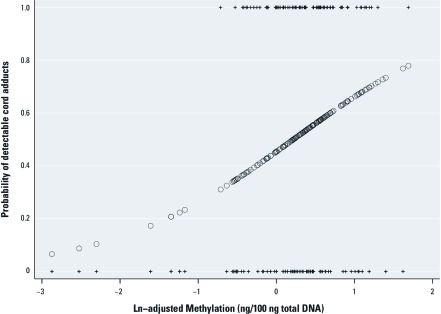
Probability of detectable BaP–DNA adducts as a function of global methylation in cord blood (based on univariate association). Circles represent the predicted probability of adduct detection based on logistic regression analyses where global methylation is an independent predictor; + indicates observed methylation at *y* = 0 for those without detectable adducts and at *y* = 1 for those with detectable adducts.

*DNA methylation and BaP–DNA adduct formation.* Newborns with detectable cord BaP–DNA adducts had higher levels of genomic methylation than did those with nondetectable adducts (odds ratio = 2.35; 95% CI: 1.35, 4.09; *p* < 0.01) (Figure 3). None of the covariates presented in Table 2 confounded this association. In this analysis, global methylation levels did not mediate or modify the relationship observed between PAH exposure and the presence of detectable adducts.

## Discussion

Among African-American and Dominican newborns in a New York City birth cohort, we found that prenatal exposure to PAHs measured using a personal air monitor during the third trimester of pregnancy was associated with lower global methylation levels measured in umbilical cord WBC DNA. We did not observe similar associations between PAHs and methylation when prenatal PAH exposure was estimated by urinary PAH metabolites, but the subset of 87 participants for whom we had metabolite data may not have provided us with adequate statistical power to evaluate this association. We also found that higher global methylation levels were positively associated with the presence of detectable adducts in cord blood, but we did not observe associations between estimates of PAH exposure [estimated using air monitors, questionnaires (diet and ETS), or metabolite levels] and detectable BaP–DNA adducts.

PAH–DNA adduct levels provide an integrated index of biologically effective dose, indicative of both exposure to PAHs from all routes (including both inhalation, dermal absorption, and ingestion) and individual metabolic differences in detoxification (Veglia et al. 2008). As we have reported previously in this cohort, BaP–DNA adducts were not significantly associated with individual measures of PAH exposure in personal air, nor were they related to ETS or an indicator of dietary PAHs based on self-reported consumption of foods known to be high in PAHs (Perera et al. 2004). In the sample of cohort participants we studied here, our results are consistent, perhaps because adducts reflect individual exposure, susceptibility to adduct formation, and repair. In this investigation, BaP–DNA adducts were measured using HPLC-F, which provides a measure that is specific to BaP adducts. However, we did not detect PAH–DNA adducts in > 50% of our samples, which limited our power to explore this association more thoroughly. Because we did not detect the theoretically probable positive association between PAH or BaP exposure and BaP–DNA adduct formation, we were unable to further explore the role that methylation plays in this biological pathway in our study sample.

Global methylation, as measured in this study, represents the overall methylation state of the genome without indicating which genomic positions are methylated. This measure can be used as a “first screen” to determine whether airborne prenatal PAH exposure levels are high enough to influence methylation status. The findings of this investigation indicate that the levels of prenatal PAH exposure experienced by our study population were associated with significant differences in global DNA methylation, independent of maternal age or parity, or child’s age or ethnicity.

Several toxicologic studies have suggested that benzo[*a*]pyrene diolepoxide (BPDE), a biologically reactive metabolite of PAH, inhibits DNA methylation. In one study, BALB/3T3 mouse cells treated with BPDE (but not BaP) had a significant inhibition of global DNA methylation (Wilson and Jones 1983). These authors hypothesized that this inhibition could be due to a variety of mechanisms, including the formation of adducts, which could, in theory, interfere with methyltransferase activity (Drahovsky and Morris 1972). In support of this theory, a recent study reported that developmental BaP exposure alters the expression of glycine *N*-methyltransferase, an enzyme involved in 1-carbon metabolism and the regulation of DNA methylation (Fang et al. 2010). In contrast, BaP-treated human breast cancer cell lines did not show reduced global methylation measured using a methyl-acceptor assay, but BaP treatment did induce gene-specific hypo- and hypermethylation (Sadikovic and Rodenhiser 2006). These authors suggested that the loss of DNA methylation is likely to occur at endogenously hypermethylated regions (e.g., genomic repetitive elements).

Relatively few epidemiologic studies have examined the effects of exposure to PAHs on DNA methylation. One such study explored the effects of PAH exposure on global methylation (estimated by measuring the methylation of LINE-1 and Alu repetitive elements) among male, nonsmoking coke-oven workers and controls in Poland (Pavanello et al. 2009). The authors reported that global methylation was higher among the coke-oven workers than nonexposed controls, whose PAH exposure was estimated based on BPDE–DNA adducts and 1-hydroxypyrene in urine. This is consistent with our finding that detectable adducts were associated with higher global methylation levels; however, we observed no association between 1-hydroxypyrene levels and global methylation in a subset of our study population. In contrast with our study, Pavanello et al. (2009) reported that PAH metabolites and adducts were correlated, which may reflect high occupational exposure to PAHs in their population of coke-oven workers, whereas our participants were exposed to lower levels of PAHs in ambient air in an urban setting.

Another study examined whether prenatal exposure to tobacco smoke, as well as passive smoke exposure in childhood and current smoking in adulthood, was associated with global methylation levels in adult blood estimated using a [^3^H]methyl acceptance assay (Terry et al. 2008). The authors reported that although prenatal tobacco exposure was associated with higher levels of methylation, childhood and concurrent smoking was associated with lower levels of methylation. In our study, neonates exposed prenatally to ETS had methylation levels similar to those of nonexposed neonates. Consistent with our report, Terry et al. (2008) observed an inverse association between smoking and global methylation when both were assessed concurrently in adults. Therefore, it may be that concurrent smoke or PAH exposure has a more pronounced effect on global methylation than past exposure. The study by Terry et al. (2008) also found lower levels of methylation among blacks compared with whites or Hispanics, which is consistent with our findings that self-identified African Americans had significantly lower methylation levels than did Dominicans.

The positive association that we observed between global DNA methylation and the presence of DNA adducts is consistent with previously published literature (Pfeifer 2006; Tretyakova et al. 2008; Zhang et al. 2005). It has been hypothesized that BPDE preferentially targets methylated CpG sites. A number of stereochemical mechanisms have been proposed to explain this apparent affinity, including that methylation alters the reactivity of the CpG dinucleotides, making them more attractive binding sites (Tretyakova et al. 2008), possibly by altering the hydrophobicity (Pirogov et al. 1998) or polarizability (Sowers et al. 1987). Studies examining the nucleotide location of BPDE adduct formation along the *p53* tumor suppressor gene have found a high correlation between adduct formation sites [which are mutation hot spots (Pfeifer et al. 2002)] and patterns of cytosine methylation within the *p53* gene (Denissenko et al. 1997). In these studies, BPDE-DNA adducts preferentially targeted guanines 3´ to methylated cytosines, and CpG methylation strongly enhanced BPDE adduct formation (Chen et al. 1998; Denissenko et al. 1997; Tretyakova et al. 2002; Weisenberger and Romano 1999). The positive association that we observed between methylation and BaP–DNA adducts was nonspecific but consistent with the findings of *in vitro* experiments.

An alternative hypothesis suggests that binding of BPDE to DNA encourages methylation of DNA by influencing DNA methyltransferase activity (Subach et al. 2006). If correct, then it is also possible that the positive association we observed between DNA methylation and BaP–DNA adducts was due to increased methylation at sites of BaP–DNA adducts. However, we could not discern the temporal sequence of methylation and adduct formation in this study.

## Conclusions

In this study we provide evidence that prenatal PAH exposure may alter global DNA methylation and that altered DNA methylation may influence BaP–DNA adduct formation. Understanding both the ability of environmental chemicals to alter methylation and the association between altered methylation and the formation of DNA adducts is critical to develop strategies to reduce disease risk. Future studies that can identify factors that influence susceptibility to PAH–DNA adduct formation and measure site-specific CpG methylation and adduct formation *in vivo* will improve our ability to understand these complex molecular relationships and biological pathways.
